# Pharmacological inhibitors of c-KIT block mutant c-KIT mediated migration of melanocytes and melanoma cells *in vitro* and *in vivo*

**DOI:** 10.18632/oncotarget.10001

**Published:** 2016-06-14

**Authors:** Christian Posch, Homayoun Moslehi, Martina Sanlorenzo, Gary Green, Igor Vujic, Renate Panzer-Grümayer, Klemens Rappersberger, Susana Ortiz-Urda

**Affiliations:** ^1^ Department of Dermatology, Mt. Zion Cancer Research Center, University of California San Francisco, San Francisco, CA, USA; ^2^ Department of Dermatology, The Rudolfstiftung Hospital, Academic Teaching Hospital, Medical University Vienna, Vienna, Austria; ^3^ School of Medicine, Sigmund Freud University, Vienna, Austria; ^4^ Children's Cancer Research Institute, St. Anna Kinderspital, Vienna, Austria; ^5^ Department of Medical Sciences, Section of Dermatology, University of Turin, Turin, Italy

**Keywords:** targeted therapy, kinase, kinome, melanoma, migration

## Abstract

Mutations in the receptor tyrosine kinase c-KIT (KIT) are frequent oncogenic alterations in melanoma and are predominantly detected in tumors of acral, mucosal, and chronically sun-damaged skin. Research indicates that melanocytes with aberrant KIT signaling can be found in the distant periphery of the primary tumor; However, it is hitherto unknown whether KIT might confer a migratory advantage, thereby enabling genetically abnormal cells to populate a distal area. In this study, we investigated the role of mutant KIT in melanocyte- and melanoma cell migration using KIT mutant lines as well as genetically manipulated murine and primary human melanocytes. Our results revealed that melanocytes, stably transduced with mutant KIT closed a gap inflicted on cell monolayers faster than wild-type controls. Similarly, KIT mutant human melanoma lines were able to populate a larger area in a 3D *in vitro* skin model compared to KIT wild type and BRAF mutant lines. Genomic profiling revealed that genes associated with increased cell-dispersal of KIT mutant variants were linked to a statistically significant up-regulation of 60 migratory genes (z-score 1.334; p=0.0001). In addition, *in vivo* experiments harnessing a mouse xenograft model of early melanoma development demonstrated rapid lateral migration of KIT mutant cells compared to respective controls. The specific kinase inhibitors imatinib and nilotinib, could abrogate this migratory advantage *in vitro* and *in vivo*. Our work suggests that KIT inhibition might help to target migratory active, KIT mutant melanoma cells, thus representing a potential strategy to reduce spread and local recurrence.

## INTRODUCTION

Melanoma is the most lethal form of skin cancer and an estimated 132.000 patients will contract this disease per annum world-wide (WHO, accessed 2015). Despite major advances in treatment of late stage skin cancers in the recent past, the issue of restricting local recurrences in early melanoma has not yet been addressed extensively [1]. Hence, and in light of the lethality of this disease, it is urgent to investigate the molecular basis for (local) melanoma spread and migration, so as to be able to improve local tumor control and potentially prevent further disease progression.

Previous studies have identified patterns of genetic alterations that are associated with distinct clinical and histological features of melanoma [2–4]. One important finding was the discovery of frequent mutations and amplifications of the receptor tyrosine kinase c-KIT (KIT) in acral, mucosal and melanomas of chronically sun-damaged skin [2, 5]. KIT acts as a *bona fide* oncogene, driving tumor cell proliferation, progression, and migration through the activation of downstream signaling cascades such as the MAP kinase and PI3K/mTOR pathways [6, 7]. Adjacent to the histologically visible *in situ* portion of such lesions, a “field effect” has been described, in which field cells were shown to occur in the wider periphery of the invasive part of the tumor [8].

Evidence indicates that these genetically abnormal field cells may be a source of local recurrences. This field effect appears to be confined to melanomas with a lentiginous growth pattern i.e. intra-epidermal growth in which melanocytes are arranged as single units within the basilar epidermis such as in melanomas affecting non-hirsute, acral skin, and melanomas of chronically sun-damaged skin, including lentigo maligna (melanoma) [4]. These melanoma types frequently have activating genetic alterations in the KIT signaling pathway, represented predominantly by mutations and amplifications of KIT itself [2, 3, 5]. Studies with multiple markers designed to detect genetic aberrations established that the number of genetic alterations are altered in a particular sequence. The finding that most mutations occurred in the invasive component, followed by the adjacent *in situ* portion, and lastly by the field cells, firmly established a genetic progression from field cells to more advanced stages and excluded the possibility that field cells represented a form of metastasis. According to this model, KIT pathway activation represents an early or even initiating event that is followed by the acquisition of additional genetic alterations required to form clinically and histologically detectable lesions [9–11].

In the present study, we investigated the role of mutant KIT in melanocyte migration. In addition we addressed the issue of whether the lentiginous growth pattern and extended field effect observed in melanomas with somatic mutations in KIT were a direct consequence of KIT pathway activation. Using human skin reconstructs grafted onto immunodeficient mice, we studied the migration and growth of genetically engineered melanocytes and melanoma cells expressing relevant KIT mutations. Additional *in vitro* experiments were undertaken so as to detect molecular changes linked to aberrant KIT signaling. In addition, we evaluated the impact of KIT inhibitors to reduce migration of KIT mutant melanocytes and regress intraepidermal melanoma progression. We present our results with reference to new insights for the tendency of certain melanoma types to recur after apparently complete excision and discuss the potential therapeutic use of KIT inhibitors to treat early melanoma.

## RESULTS

### KIT mutant cells have a migratory advantage over wild type melanocytes

**Figure 1:**
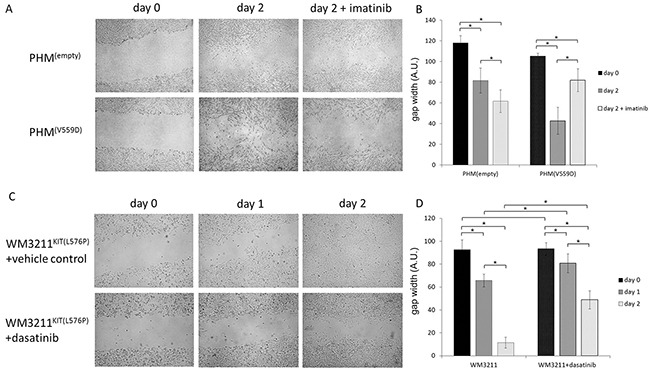
Mutant KIT confers a migratory advantage **A.** Representative pictures of wound-healing assays using primary human melanocytes (PHM) with the indicated genetic modifications, and **B.** statistical analyses comparing differences in gap widths. KIT mutant cells were able to close the artificial wound faster than non-mutant cells. This migratory advantage was reversed by using the specific KIT inhibitor imatinib at a concentration of 100nM (n>5; 2-sided student t-test; * p<0.05; error bars indicate the standard deviation). **C, D.** Similarly to results in KIT mutant PHM, the cell line WM3211^KIT(L576P)^ also showed reduced migratory capacity when incubated with specific KIT inhibitor dasatinib at a concentration of 10nM compared to vehicle control conditions (n>5; 2-sided student t-test; * p<0.05; error bars indicate the standard deviation).

To investigate the migratory capacity of KIT mutant cells, we stably transduced primary human melanocytes (PHM) with mutant KIT^(V559D)^ or empty vector controls. Cells were grown as a monolayer to which a wound was introduced by scratching, and the rate of wound closure was monitored microscopically. Results showed that cells bearing mutated KIT could bridge the gap as early as in 48h, whereas the gap introduced into a control cell monolayer remained open (Figure 1AB). To further differentiate whether wound closure was accelerated due to increased proliferation or the induction of migration, PHM variants were pretreated with mitomycin C, a potent inhibitor of cell proliferation [12]. Mitomycin C reduced the rate of wound closure in both KIT^(V559D)^ and empty vector control PHMs indicating that proliferation adds to faster wound closure in mutant cells. However, similar to our initial results with untreated cells, mitomycin C treated KIT^(V559D)^ mutant PHMs closed the artificial wound faster than their respective empty vector controls (Supplementary Figure S1).

Hence, it appeared that expression of the KIT mutant gene accelerated wound healing, a function of cell migration, and it would be interesting to determine whether anti-KIT inhibitors might arrest this accelerated cell spread.

### KIT inhibitors slow KIT mutant cell migration

To verify that our previous observation of accelerated gap closure was due to the mutant KIT gene, we used the kinase inhibitors imatinib and dasatinib for targeting the KIT receptor. The aforementioned variants of PHMs, but also human KIT mutant melanoma cell line WM3211^KIT(L576P)^ were used. Adding the kinase inhibitor imatinib at a concentration of 100nM selectively impeded the accelerated cell dispersal in KIT mutant PHMs (Figure 1AB). Pharmacological KIT inhibition with dasatinib at a concentration of 10nM also reduced gap closure of the KIT mutant melanoma cell lines WM3211^KIT(L576P)^ (Figure 1CD).

Results indicated that KIT inhibitors could block accelerated cell dispersal in KIT mutant cells, further supporting an important role of KIT signaling for cell migration. It would thus be interesting whether such an advantage in cell-spread was limited to culture dishes or could also be observed in an *in vitro* 3D model of human skin.

### KIT mutants populate a larger area on devitalized human dermis compared to wild type PHMs and BRAF mutant melanoma cells

**Figure 2:**
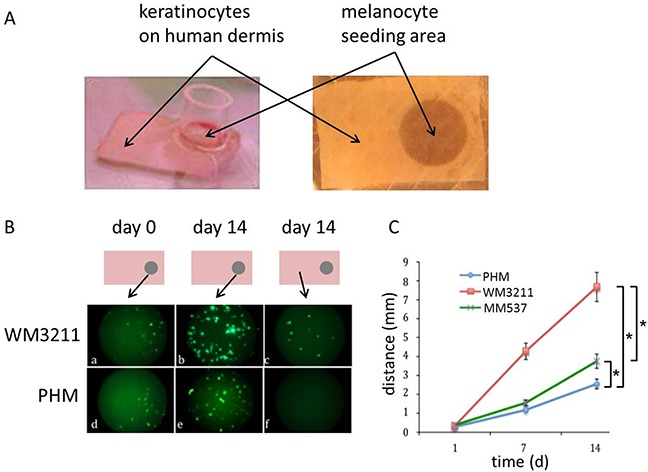
KIT mutant cells migrate faster on human dermis than wild type human melanocytes and BRAF mutant melanoma cells **A.** Representative picture displaying the generation of human skin reconstructs in vitro: GFP expressing PHMs or melanoma cell lines mixed with human keratinocytes (ratio 1:6) were seeded in a cylinder on one edge of devitalized human dermis. The rest of the graft was covered with human keratinocytes only. **B.** Schematic drawing of skin reconstructs indicating the location of the representative microscopic pictures: GFP expressing, KIT mutant cells were able to populate a greater area on devitalized human dermis after 14 days of incubation compared to wild type PHMs or BRAF mutant melanoma cells (c,f). **C.** Statistical analyses of distance migrated over time by GFP expressing cells (n>3; 2-sided student t-test * p<0.05; error bars indicate the standard deviation).

Our next set of experiments was performed in an organotypic melanocyte-keratinocyte co-culture. Briefly, combined human keratinocytes and GFP-expressing melanoma cell lines harboring mutations in either KIT^L576P^ (WM3211), B-RAF^V600E^ (MM537) or PHMs being wild type for these mutations were seeded onto devitalized human dermis at a 6:1 ratio. GFP expressing cells were added on one of the edges of the insert. Cell dispersal was followed over time with microscopy. We found that while melanoma cell lines expressing mutant KIT migrated over 7mm away from the seeding area after 2 weeks of 3D culture, BRAF mutant melanoma cells and wild type melanocytes only migrated less than 4mm from the seeding site at 2 weeks (Figure 2; 2-sided student t-test; p<0.05).

Findings using our *in vitro* model of early melanoma support the above observations that mutant KIT fuels cell dispersal *in vitro*. We next wanted to know which molecular mechanisms can be linked to activated, mutant KIT and whether such signaling events might help to refine the observed phenotype.

### Mutant KIT affects signaling cascades important for posttranslational modification and migration

**Figure 3:**
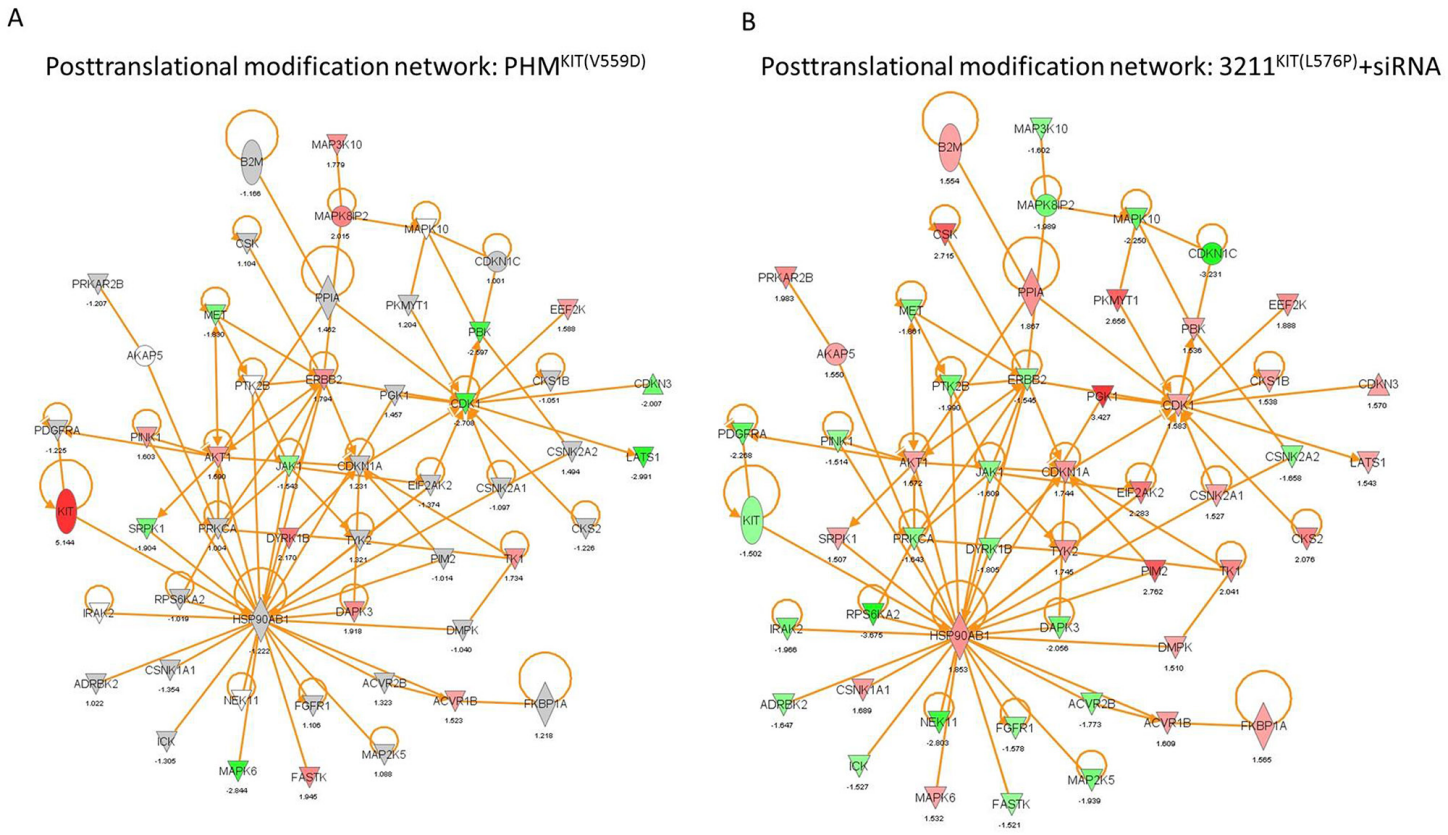
KIT regulates genes linked to posttranslational modification **A.** Posttranslational modification (PTM) network of PHMs bearing mutant KIT^(V559D)^ compared to empty vector control cells. **B.** PTM network of WM3211^KIT(L576P)^ cells with siRNA mediated reduction of KIT expression compared to scramble control cells. (red: upregulation, green: downregulation).

In our next set of experiments we investigated signaling changes in KIT^(V559D)^ mutant versus control vector bearing PHMs using a TagMan based kinome array panel assaying for 828 specific kinases and kinase-related genes, including 10 endogenous control genes. Analyses revealed that 246 genes were at least 1.5-fold differentially expressed in KIT^(V559D)^ mutant versus empty vector controls. GO annotation analysis revealed that top molecular and cellular functions affected were “Post-Translational Modification” (143 molecules, p<0.0001) (Figure 3A), “Cell Signaling” (79 molecules, p<0.0001) and “Cell Death and Survival” (138 molecules, p<0.0001) (Supplementary Table S1–S3). The GO term “Migration” was also among the top molecular and cellular functions affected (60 molecules, p<0.0001). The z-score for the GO annotation “Migration” was calculated with 1.334 (p=1.25E-7) indicating a functional increase of genes involved in cell movement, migration and invasion.

In a second set of experiments, we transiently silenced KIT in the KIT mutant cell line WM3211^KIT(L576P)^. Out of 828 target genes, 133 were differentially expressed after KIT silencing in WM3211 cells. Similar to results obtained from PHMs expressing mutant KIT, the top regulated molecular functions were “Post-Translational Modification” (Figure 3B; 80 molecules, p<0.0001), “Cell Death and Survival” (78 molecules, p<0.0001) and “Cell Signaling” (43 molecules, p<0.0001) (Table S4–6). We also found significant reduction of molecules mapping to the GO term “Cellular Movement” (z-score=−2.23; p=2.56E-02) when KIT was knocked down in WM3211^KIT(L576P)^ cells. Additionally, most targets in the “Post-Translational Modification” network mapped to genes that have been described in migration (Supplementary Figure S2). As previously described, additional pathway analyses using our kinome array data showed the regulation of the PI3K/mTOR and MAPK pathways when KIT signaling was genetically manipulated (Supplementary Figure S3–S6) [13–15].

Data further established the importance of mutant KIT for cell homeostasis, survival and cell migration *in vitro*. Following up on these findings we studied if KIT mutations could also fuel enhanced cell dispersal *in vivo*.

### KIT mutant Melan A cells show features of migration in an *in vivo* model of human skin reconstructs

To investigate if KIT mutant cells can be used to study *in vivo* migration, we established a mouse model of human skin reconstructs mimicking early melanoma progression (Supplementary Figure S7).

**Figure 4:**
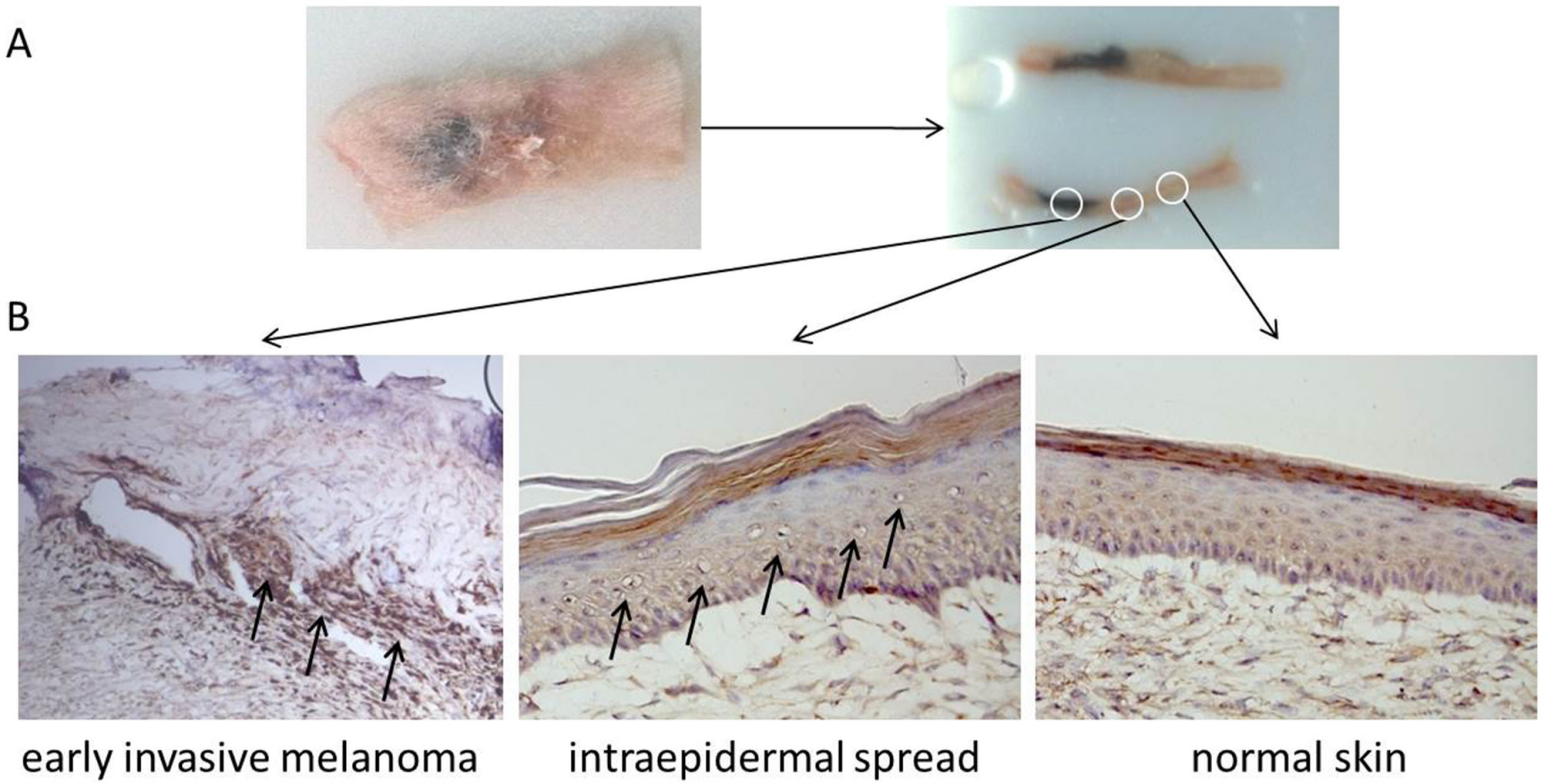
KIT mutant skin reconstructs share histological features of early human melanoma **A.** Harvested, flash frozen and embedded human skin reconstruct graft 4 weeks after transplantation onto the back of a NOD/SCID mouse. **B.** Histological examination revealed early invasive melanoma of the heavily pigmented KIT^(V559D)^ mutant Melan A cells in the seeding area, intra-epidermal spread of melanocytes in close proximity to the seeding area and normal skin in the distal periphery of the graft.

Due to the limited life span of PHMs, we used mouse Melan A cells for our *in vivo* models. To allow for best possible comparability of *in vitro* and *in vivo* findings we investigated signaling changes due to mutant KIT^(V559D)^ in Melan A cells. Similarly to the kinome array results using PHMs, KIT^(V559D)^ mutant Melan A cells also showed an increase of MAPK and PI3K/mTOR signaling compared to parental empty vector bearing cells (Supplementary Figure S8). For our *in vivo* model we used a silicon cylinder to seed a mixture of primary human keratinocytes and Melan A cell variants at a defined area onto human dermis. The remainder of the graft was covered with primary human keratinocytes only. Constructs were then cultured for 7 days and transplanted onto the back of immuno-compromised NOD/SCID mice (Supplementary Figure S7). After establishing this model, we histologically investigated the skin graft for the heavily pigmented KIT^(V559D)^ transduced Melan A cells. Macroscopic evaluation and histology of KIT mutant grafts 4 weeks after transplantation revealed early invasive melanoma at the seeding area, intra-epidermal spread of melanocytes close to the seeding area and normal skin at the distal end of the graft (Figure 4AB), features reflecting early melanoma development.

Data indicated that our model of early melanoma development could be used to study the lateral expansion of melanoma cells *in vivo*. Therefore we investigated the impact of KIT inhibitors to affect distal spread of KIT mutant cells in our xenograft model.

### Specific KIT inhibitors reduce migration of KIT mutant melanoma cells *in vivo*

**Figure 5:**
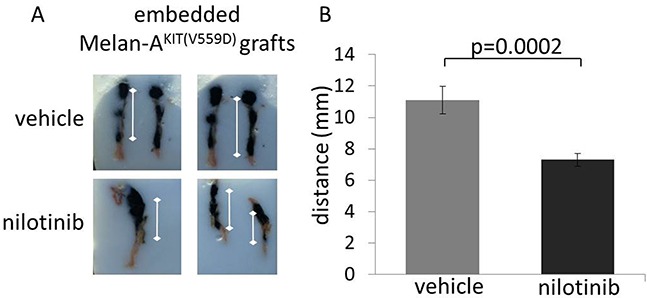
The early use of pharmacologic KIT inhibitors prevents KIT mutant melanocyte migration *in vivo* **A.** Representative, embedded vehicle treated and nioltinib treated human skin reconstruct grafts. **B.** Macroscopic evaluation (measured from the center of the seeding area to the distal end of the tumor) revealed that a greater area of the skin reconstruct was colonized by the heavily pigmented KIT^(V559D)^ mutant Melan A cells in vehicle treated mice compared to nilotinib treated mice (n=4 for each group, 2-sided student t-test: p=0.0002).

Using our *in vivo* xenograft model of early melanoma, we tested the systemic administration of the small molecule KIT inhibitor nilotinib to prevent melanoma development and horizontal spread. Nilotinib is a potent receptor tyrosin kinase inhibitor with activity in the low nanomolar range and is more potent for *in vivo* KIT inhibition compared to imatinib [16]. As described before, skin reconstructs bearing KIT^(V559D)^ mutant Melan A cells were transplanted onto the back of immuno-compromised NOD/SCID mice. Two days post transplantation mice were systemically treated with the KIT inhibitor nilotinib at a dosage of 75mg/kg/day by oral gavage. After 3 weeks of treatment, skin reconstructs were collected and analyzed. Data revealed a reduction of horizontal KIT mutant cell-spread by 34% (n=4; 2-sided student t-test; p=0.0002) in nilotinib treated mice compared to the vehicle control treated group (n=4) (Figure 5AB). No side effects of the treatment were noticed.

Hence, based on our combined results we could demonstrate both *in vitro* and *in vivo* that acceleration of melanoma cell spread due to increased KIT activity could be arrested with specific kinase inhibitors. The implications of these findings are discussed below.

## DISCUSSION

KIT is a receptor tyrosine kinase encoded by the proto-oncogene c-kit and is essential for the survival, growth, differentiation, migration, and homing of melanoblasts to the basal dermis [9–11]. Gain-of-function mutations in KIT have been found in several cancers. In melanoma patients, acral, mucosal and melanomas of chronically sun-damaged skin have been found to frequently harbor genetic aberrations affecting KIT [2, 5].

In the present work, we demonstrated that mutant KIT signaling in melanoma cells and melanocytes, a condition affecting 5-7% of all melanomas, accelerated their horizontal dispersion from the core lesion to the surrounding healthy appearing skin. By applying kinome-centered arrays, we could refine this dispersal process into one of increased survival of cells and intentional migration with significantly upregulated migratory genes in the situation of overexpressed KIT. In addition, we also demonstrated a novel inhibitory function for the pharmacological agents imatinib, dasatinib and nilotinib, currently used to treat metastatic KIT mutant melanoma patients, to block this migration. This latter observation, whereby melanomas could additionally be targeted for their migratory capacity, might extend our ordnance to treat melanoma. The clinical importance of KIT mutations in melanoma and the role of activated KIT in disease spread and progression relates to the fact that drugs for inhibition of its kinase activity such as imatinib, dasatinib and nilotinib are readily available and are already used successfully in patients with advanced metastatic melanoma and other cancers [17–20].

The primary treatment of melanoma is surgical excision. However, even when melanoma is excised with sufficient safety margins disease can still recur. Melanomas of chronically sun-damaged skin and those found on the palms and soles are often poorly circumscribed and have a high tendency to recur despite apparently clear surgical margins. To date, local recurrences are proposed to be of two kinds: re-growth of an incompletely resected primary tumor and local metastasis, each with different prognostic implications [21, 22]. The National Comprehensive Cancer Network recommends that cutaneous melanoma be excised with a margin of clinically normal appearing skin of 0.5 to 2cm, depending on the thickness of the primary melanoma. The recommendations are based on clinical trials that did not distinguish the two proposed mechanisms for local recurrence. The current recommended safety margin for the treatment of *in situ* melanoma (lentigo maligna) is 0.5cm. However, several studies show that 0.5cm margins are frequently inadequate [23–26].

Recently, a new precursor phase of acral melanoma, the field effect, was identified, suggesting that genetic aberrations in a subset of migratory cells located in close proximity to the invasive portion of a melanoma might be the source of local recurrence [8]. The lentiginous growth patterns of these lesions, the frequent genetic aberrations in KIT observed in such tumors, and the migratory advantage of KIT activated melanocytes are the rational that KIT pathway activation might confer an increased (intraepidermal) migratory phenotype of melanoma cells [7, 27, 28]. The critical role of KIT in melanocyte survival and migration is further illustrated by naturally occurring inactivating mutations in various developmental disorders where they result in amelanotic congenital patches of white skin [7, 11]. Data presented herein not only further support that mutant KIT induces motility of melanocytes and melanoma cells *in vitro,* but also indicate an *in vivo* migratory advantage of KIT mutant melanoma that might, at least in part, contribute to the field effect and high rate of local recurrence in KIT activated melanoma. Most importantly, this effect can be reversed by the early use of pharmacological KIT inhibitors. The use of mitomycin *in vitro* allowed us to reduce potential effects of proliferation when assessing the migratory capacity of cells. However, we cannot fully exclude that a reduction in cell viability by kinase inhibitors and off-target effects might contribute to the observed reduction of cell dispersal *in vivo*.

Signaling downstream of KIT is complex. Yet, the activation of the MAP kinase and PI3K/mTOR pathways appear to be an important mechanism by which constitutive KIT activation contributes to the observed phenotype of cells [13, 15]. Most KIT alterations occur in exon 11, including point mutations in position 559, which was used in the present study [3]. Exon 11 encodes the juxtamembrane domain, with mutations leading to KIT dimerization in the absence of the ligand stem cell factor (SCF) resulting in constitutive activation [17, 29]. Further studies are necessary to investigate whether mutations in other frequently mutant domains of KIT, such as the kinase domain I or II, might also result in a similar cell phenotype and if the administration of specific kinase inhibitors might be beneficial.

To conclusion, our study identified mutant KIT as a critical component of a mechanism for migration and spread of melanoma cells. It would be important to determine whether KIT signaling might also play a role in disease progression in other solid tumors, including gastrointestinal stromal tumors, where KIT mutations are found frequently [17–20, 30]. Furthermore, it will be required to investigate if findings in our model system hold true in melanoma patients. Still, our results indicated that KIT inhibitors might help to reduce local recurrence of KIT-activated melanoma by reducing migration in addition to reducing viability and might thus be considered for the early treatment of these tumors. Similarly, extending the application of KIT inhibitors to the treatment of other malignancies has the potential to improve patient care.

## MATERIALS AND METHODS

### Cell lines and cell culture

Cell lines WM3211, MM537, and Melan A were available in our cell repository at the University of California, San Francisco and maintained in RPMI 1640 media supplemented with 10% (vol/vol) FBS. Primary human melanocytes (PHM) and primary human keratinocytes were extracted from left-over foreskin tissue. Tissue was digested overnight in 50% (vol/vol) dispase and M254 medium (invitrogen, M-254-500) for melanocytes and Keratinocyte Serum free medium (SFM) with supplements (gibco, 17005-042). The epidermis was peeled off gently and digested with 0.05% (vol/vol) trypsin for 5min at 37°C. Trypsin was neutralized with equivalent volume of DMEM (Gibco, 11995) supplemented with 10% (vol/vol) FBS and 1x penicilin/streptomycine (Gibco, 15140). After shaking the cell suspension for 3min, cells were spun down at 1000RPM for 5min, resuspended in M254 medium for melanocyte extraction and grown for 4-5 doublings. For keratinocyte extraction the cell suspension was gently shaken for 30 seconds and the cell pallet resuspended in keratinocyte SFM. All experiments in PHM were performed in a pool of PHM, derived from 6 different donors. Samples were obtained with the approval of the University of California San Francisco, Institutional Review Board (IRB#12-09483). All cell lines were incubated at 37°C under 5% CO_2_.

### Transduction

Lentiviral transduction was achieved by first cloning the KIT cDNA into the Gateway entry vector pENTR/D-topo. pENTR/D-topo-KIT was subjected to site-directed mutagenesis to generate a variety of known KIT mutants which were then validated by Sanger sequencing. KIT cDNAs in pENTR were cloned into the Gateway cloning-enabled destination vector gFG12. Green Fluorescent Protein (GFP) was co-expressed with the different transgenes and used as a transduction efficiency reporter. After lentiviral transduction, cells were grown for 2 weeks followed by cell sorting facilitating GFP intensity on a FACS Aria II cell sorter. Sorted cells were then grown continuously in the respective conditions and monitored for GFP expression at least once a week.

### Inhibitors, viability and migration assays, organotypic co-culture

All inhibitors used in the study were purchased from Selleck Chemicals. Viability assays were carried out at least in triplicates. Cell migration was assayed with a monolayer wound-healing assay. Cells were seeded into 6-well culture dishes and incubated for 24h for complete attachment. At least two scratches with a 1ml pipette tip were made for each well and images were taken at indicated time points. Indicated conditions were pre-incubated with mitomycin C at a concentration of 10μg/ml for 1h. Primary human keratinocytes and the respective melanoma and melanocyte cell vatiants were pipetted onto devitalized human dermis at a ratio of 6:1, submerged in media and incubated at 37C 5% CO2 for 72 hours. Then culture was switched to medium containing 1.2mM Ca++ for 16 hours for keratinocyte differentiation. Cultures were then lifted to air by removing medium from transwells and maintained at 37C. In order to facilitate the measurement of melanocyte migration keratinocytes were plated first, followed by GFP co-expressing melanoma cell variants. Migration was followed over time with microscopy.

### Kinome array assay

Kinome array analyses were performed as previously described [31]. Total RNA from cultured cells was extracted using the miRNeasy® Mini Kit (Qiagen, 217004). 2.4 μg DNase-treated total RNA was reverse transcribed to cDNA by using the SuperScript Vilo cDNA Synthesis Kit (Life Technologies, 4453650) as described by the Early-Access TaqMan® OpenArray® Pathway Panels User Guide. cDNA was applied to the TaqMan® Kinome panel (Life Technologies, 4467775). Data was collected with a quality score cut-off of 300 and differential expression determined by Data Assist software (Life Technologies). 828 specific assays specific to kinase and kinase-related genes are identified including 10 endogenous control genes in quadruplicate. Kinome arrays were performed in triplicates.

### Human skin reconstructs, xenografts

Xenograft studies were performed in NOD/SCID interleukin-2 receptor gamma chain null mice. Grafts were prepared *in vitro* by seeding a monolayer of human primary keratinocytes on devitalized human dermal substrate. In a silicon cylinder defined area, genetically modified melanocytes (Melan A cells) were seeded. Cells were allowed to attach for 6 hours forming a sharply defined melanocyte seeding area. Then the silicon cylinder was removed and excess cells were gently washed off with medium. After 7 days in culture with media changes every other day, xenografts were transplanted to the back of mice. For inhibitor treatment studies, mice were grafted with KIT mutant reconstructs and randomly assigned to either the treatment or vehicle control group. Nilotinib was administered by oral gavage 2 days post transplantation at a dosage of 75mg/kg/day 5 days a week for 3 weeks. All animal studies were approved by IACUC/LARC of the University of California San Francisco (AN086990).

### Immunoblots

Cells were plated in 6 well plates 24h prior to protein extraction. Cells were washed with phosphate buffered saline (PBS) and lysed using radio-immunoprecipitation (RIPA) buffer [150 mM NaCl, 1% (vol/vol) Nonidet P-40, 0.5% (wt/vol) sodium deoxycholate, 0.1% (wt/vol) SDS] in 50 mM Tris·HCl (pH 8.0) supplemented with 1x protease and phosphatase inhibitors (78442; Pierce). Protein concentrations were determined using the BCA Protein Assay kit (23235; Pierce). Total protein in 1×Laemmli buffer with 10% 2-mercaptoethanol was separated by SDS/PAGE, transferred for 1 h to a PVDF membrane (IPVH00010; Millipore) by electro blotting with 20% (vol/vol) methanol, and blocked for 1 h in 5% (wt/vol) dry milk/Tris-buffered saline (TBS)/0.1% (vol/vol) Tween-20. Membranes were incubated overnight at 4°C with primary antibodies following incubation with horseradish peroxidase-conjugated secondary antiserum for 1 h and developed using enhanced chemiluminescence [32106 (Pierce) or 64 – 201BP (Millipore)]. β –Actin protein expression served as a loading control. P-ERK (4370), p-AKT (4060), and p-S6 (4857) were obtained from Cell Signaling Technology, β –Actin was purchased from Sigma Aldrich.

### Statistical analyses, GO annotations, pathway analyses

mRNA expression values from the TagMan Kinome array were calculated and compared using the Data Assist™ software (Thermo Scientific). GO annotations and pathway analyses were carried out using the Ingenuity Pathway analysis software version 21901358. A p-value of <0.05 was considered significant.

## SUPPLEMENTARY FIGURES AND TABLES


