# Supplementary material for: Pharmacological inhibitors of c-KIT block mutant c-KIT mediated migration of melanocytes and melanoma cells in vitro and in vivo
Source: Oncotarget. 2016 Jun 14;7(29):45916–25. doi: 10.18632/oncotarget.10001 (PMC5216770; doi:10.18632/oncotarget.10001)
Supplement: Supplementary file 1 [file oncotarget-07-45916-s001.pdf]

Pharmacological inhibitors of c-KIT block mutant c-KIT mediated migration of melanocytes and melanoma cells *in vitro* and *in vivo*

SUPPLEMENTARY FIGURES AND TABLES

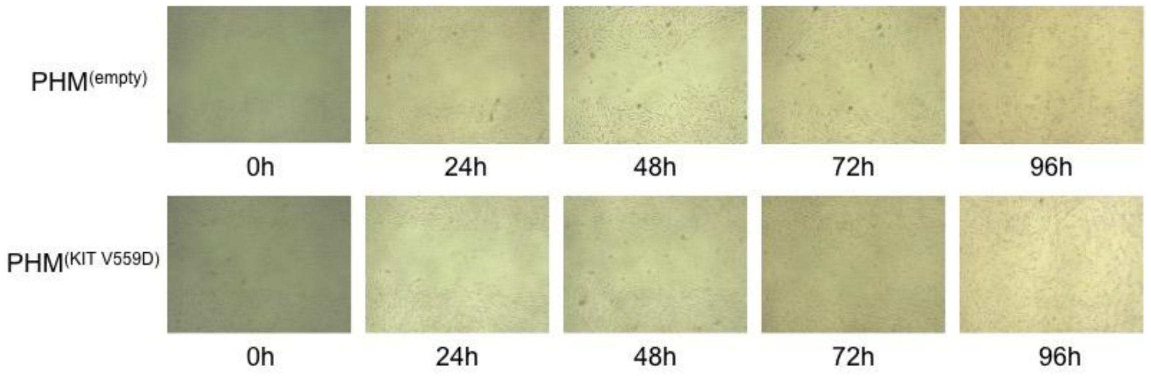

Supplementary Figure S1: Representative pictures of wound healing assays after preincubation of cells with the proliferation inhibitor mitomycin. KIT$^{(V559D)}$ mutant PHMs close the artificially inflicted gap in the cell monolayer faster than empty vector bearing PHMs (n>5).

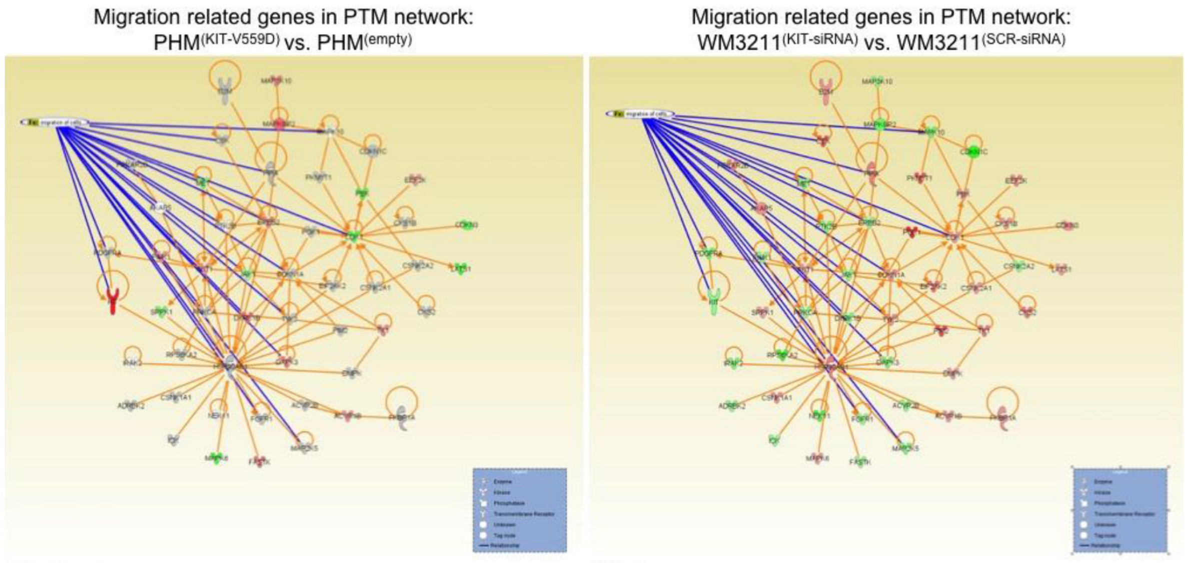

Supplementary Figure S2: Genes related to migration in the posttranslational modification (PTM) network comparing primary human melanocytes bearing either KIT$^{(V559D)}$ or empty vector controls or the KIT mutant melanoma cell line WM3211 transfected with either KIT-siRNA or SCR-siRNA.

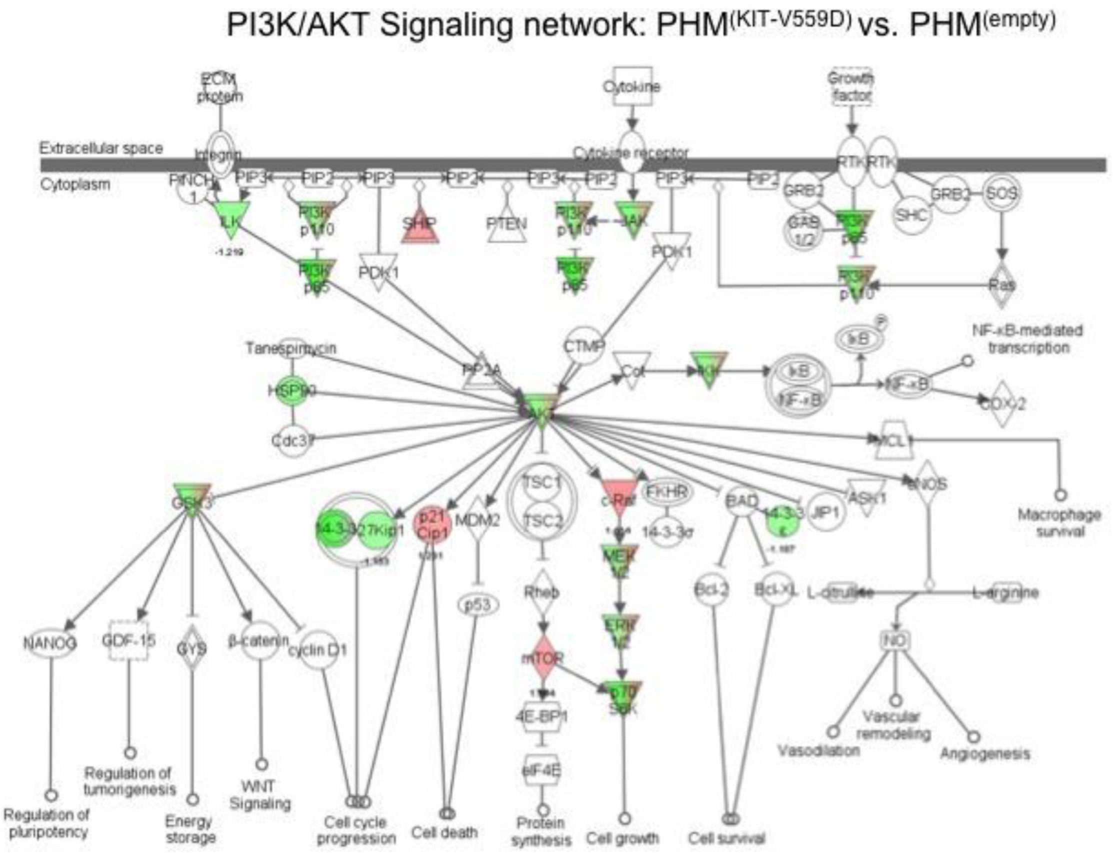

Supplementary Figure S3: Regulated signaling mediators in the PI3K/mTOR pathway of primary human melanocytes (PHM) bearing mutant KIT$^{(V559D)}$ compared to empty vector control cells.

PI3K/AKT Signaling network: WM3211$^{(KIT\text{-}siRNA)}$ vs. WM3211$^{(SCR\text{-}siRNA)}$

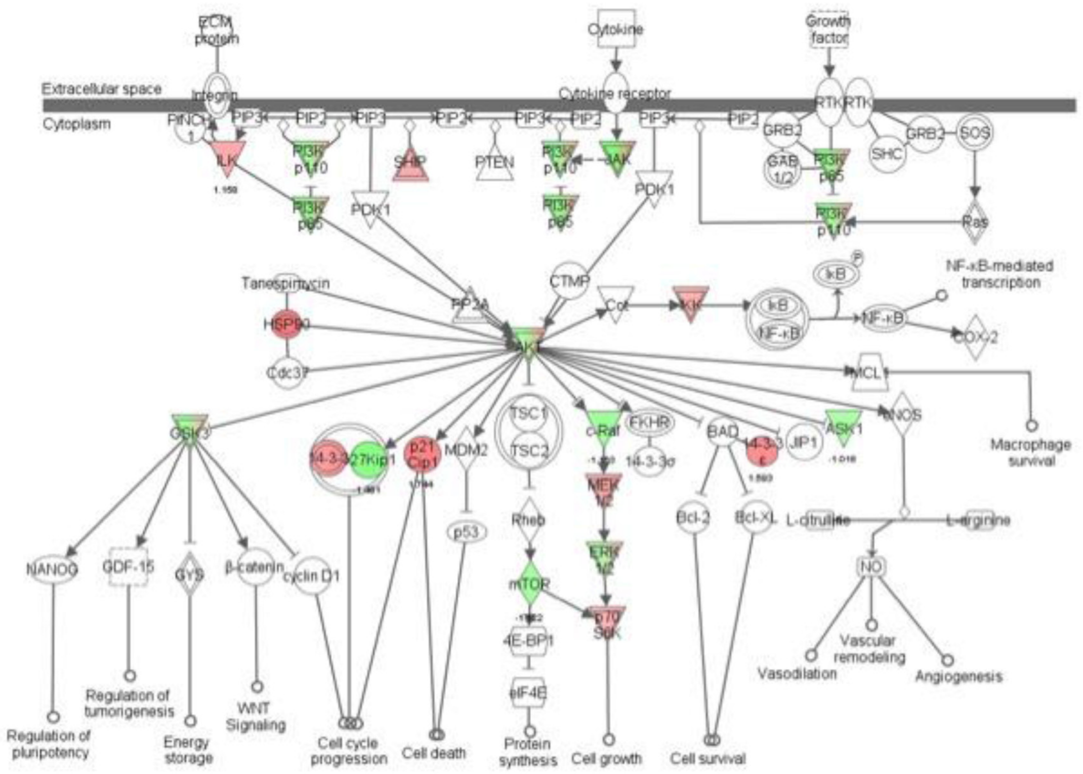

Supplementary Figure S4: Regulated signaling mediators in the PI3K/mTOR pathway of WM3211 cells with siRNA-mediated reduction of KIT compared to scramble control cells.

ERK/MAPK signaling network: PHM$^{(KIT-V559D)}$ vs. PHM$^{(empty)}$

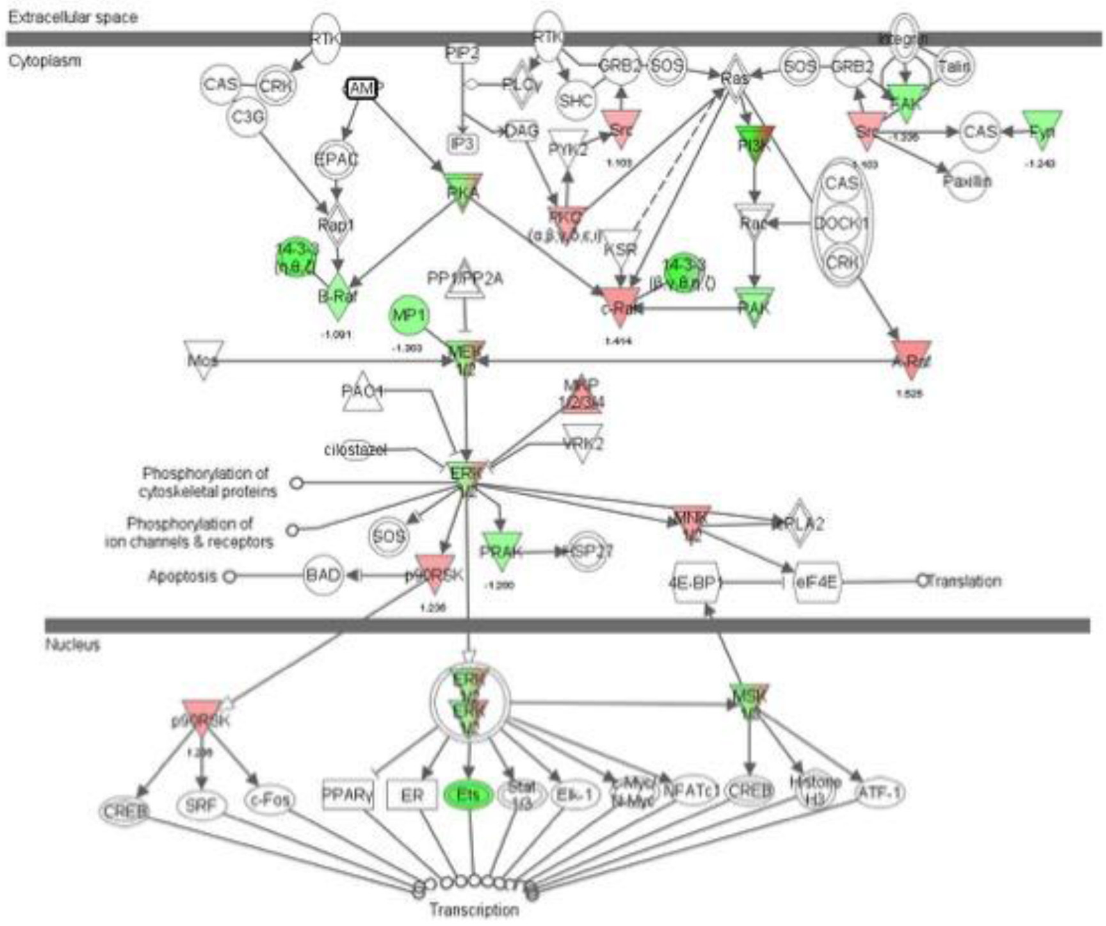

Supplementary Figure S5: Regulated signaling mediators in the MAPK pathway of primary human melanocytes (PHM) bearing mutant KIT$^{(V559D)}$ compared to empty vector control cells.

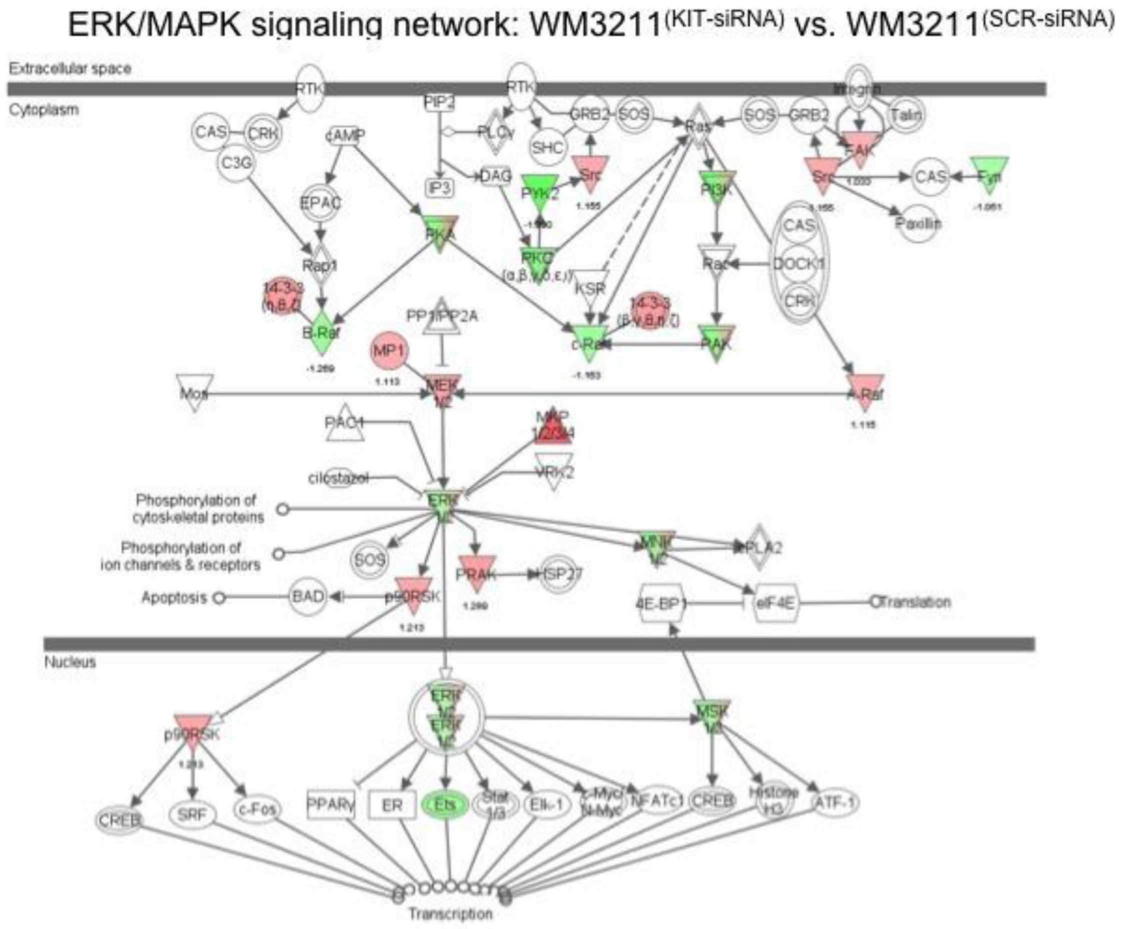

Supplementary Figure S6: Regulated signaling mediators in the MAPK pathway of WM3211 cells with siRNA mediated reduction of KIT compared to scramble control cells.

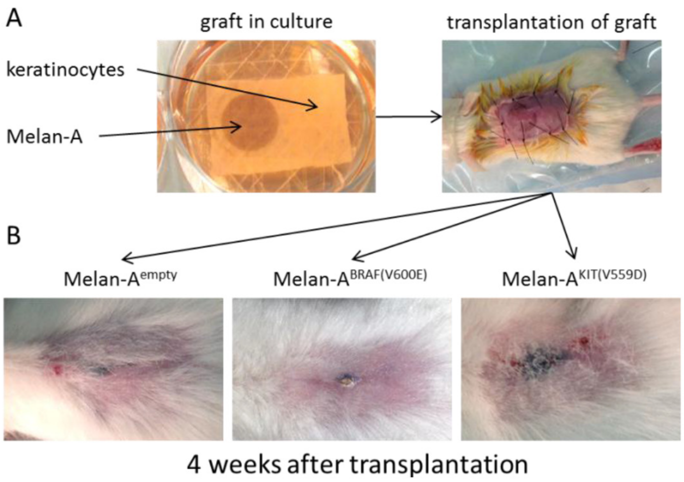

Supplementary Figure S7: KIT mutant melanocytes populate human skin reconstructs *in vivo*. A. Schematic picture of human skin reconstructs with the circular melanocyte seeding area in culture and post transplantation onto the back of NOD/SCID mice. **B.** In vivo image of immortalized Melan-A cells bearing the indicated genetic mutations 4 weeks after transplantation.

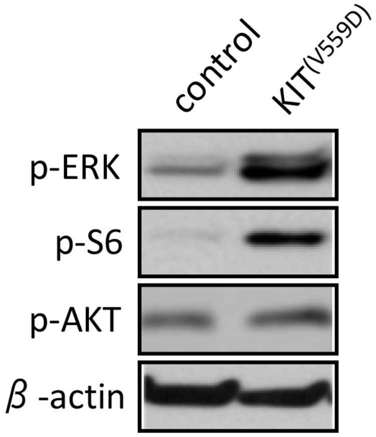

Supplementary Figure S8: Immunoblot of Melan A cells transduced with either empty vector control or mutant KIT$^{(V559D)}$ revealed induction of MAP kinase and PI3K/mTOR signaling evidenced by increased levels of p-ERK and p-S6.

Supplementary Tables S1-S3: Data of primary human melanocytes transcued with mutant KIT$^{(V559D)}$ compared to empty vector control cells using the ingenuity pathway analyses tool

PHM$^{(KIT-V559D)}$ vs. PHM$^{(empty)}$	
**Top molecules up-regulated**	
**Molecules**	**Exp. Value**
CSNK1D	30.365
KIT	5.144
CHKA	4.497
ADCK3	3.378
PIK3C2B	3.281
MOB3C	2.996
CDK18	2.876
PKD1	2.659

Top molecules down-regulated	
**Molecules**	**Exp. Value**
MAP3K2	-4.413
BUB1B	-4.082
MST4	-4.003
SRP72	-3.116
STK39	-3.036
LATS1	-2.991
MAPK6	-2.844
CDK1	-2.708

Top molecular and cellular functions		
**Name**	**p-value**	**# Molecules**
Post-Translational modification	$1.54^{-108} – 9.57^{-5}$	143
Cell signaling	$8.34^{-29} – 4.07^{-4}$	79
Cell Death and Survival	$9.28^{-25} – 6.59^{-4}$	138
Cell cycle	$2.74^{-22} – 6.54^{-4}$	93
Amino acid metabolism	$2.12^{-21} – 2.68^{-6}$	28

Supplementary Tables S4-S6: Data of KIT mutant WM3211 cells with siRNA mediated reduction of KIT compared to scramble control cells using the ingenuity pathway analyses tool

WM3211$^{(KIT\text{-}siRNA)}$ vs. WM3211$^{(SCR\text{-}siRNA)}$

Top molecules up-regulated

Molecules	Exp. Value
CSNK1D	5.040
ERNA5	4.350
HPRT1	4.152
CHKA	3.502
PGK1	3.427
TFRC	3.018
CNKSR2	2.809
HK2	2.791

Top molecules down-regulated

Molecules	Exp. Value
EFNA1	-4.764
PIK3IP1	-4.608
RPS6KA2	-3.675
CDKN1C	-3.231
NEK11	-2.803
PDK4	-2.766
DCLK2	-2.435
CAMK2N1	-2.336

Top molecular and cellular functions

Name	p-value	# Molecules
Post-Translational modification	1.2^{-60} – 2.81^{-3}	80
Cell death and survival	1.46^{-19} – 2.77^{-3}	78
Cell signaling	3.34^{-16} – 2.01^{-3}	43
Amino acid metabolism	1.49^{-15} – 3.78^{-5}	18
Small molecule biochemistry	1.49^{-15} – 2.36^{-3}	39
